# Evaluating the Effectiveness of an Intelligent mHealth Intervention for Child Unintentional Injury Prevention: Protocol for a Cluster Randomized Controlled Trial

**DOI:** 10.2196/76195

**Published:** 2025-07-18

**Authors:** Yang Yuan, Yuying Jing, Jiyu Li, Jieyi He, David C Schwebel, Peishan Ning, Zhenzhen Rao, Li Li, Guoqing Hu

**Affiliations:** 1 Department of Epidemiology and Health Statistics, Xiangya School of Public Health, Central South University, Public Health Bldg, 172 Tongzipo Road, Changsha, 410013, China, 86 15211051690; 2 Clinical Trials Center, The Affiliated Hospital of Guizhou Medical University, Guizhou, China; 3 Department of Psychology, University of Alabama at Birmingham, Birmingham, AL, United States; 4 Hunan Provincial Key Laboratory of Philosophy and Social Sciences of Urban Smart Governance, Changsha, China

**Keywords:** unintentional injury prevention, intelligent mobile health, cluster randomized controlled trial, children, China’s National Basic Public Health Service Program

## Abstract

**Background:**

Unintentional injury is a leading cause of childhood morbidity and mortality worldwide. In China, real-world implementation of child injury prevention efforts remains inadequate due to constrained workforce capacity and a lack of operational frameworks.

**Objective:**

This study aims to assess the effectiveness of a mobile health (mHealth) intervention, the Intelligent Child Unintentional-Injury Reduction & Education (iCURE) project, embedded within China’s National Basic Public Health Service Program. The intervention relies on a WeChat (Tencent) service account for caregivers and a web-based platform for health care providers to deliver standardized unintentional injury prevention strategies for young children. Key features of the program include interactive questions and answers, injury risk assessment with instant feedback, a tailored injury prevention knowledge disseminator, and regular reminders to caregivers.

**Methods:**

A double-blind, 12-month follow-up, cluster randomized controlled trial will be implemented in Changsha, Hunan Province, China. Caregivers of children aged ≤5 years will be recruited. Randomization will be conducted at the street or town level. The control group will receive routine safety education, while the intervention group will receive both routine safety education and the iCURE mHealth intervention focused on unintentional injury prevention and delivered via WeChat. Data will be collected at baseline and every 3 months during the study period. The primary outcome is 12-month incidence of unintentional injuries among children, including minor injuries and as reported by caregivers. Secondary outcomes include children’s injury risk level and caregiver supervision behaviors assessed using a standard questionnaire. Data analysis will be conducted using generalized linear mixed models with a Poisson link function and generalized estimating equations to assess the effectiveness of the iCURE intervention, following intention-to-treat principles. Sensitivity analyses will be conducted with per-protocol principles and excluding participants with missing primary outcomes.

**Results:**

As of May 2025, a total of 6701 participants have been successfully enrolled and baseline data were collected for all participants. Of those enrolled, 87.2% (5842/6,701) completed the first follow-up assessment.

**Conclusions:**

This trial will examine the effectiveness of an intelligent mHealth intervention for child unintentional injury prevention building on China’s National Basic Public Health Service Program. If successful, the iCURE intervention may provide a cost-effective strategy for child injury prevention in low- and middle-income countries.

## Introduction

Unintentional injuries remain a leading cause of morbidity and mortality among children worldwide [1]. In 2021, approximately 200,000 children younger than 5 years of age died globally from injuries and 38.7 million experienced nonfatal injuries [2]. In China, where the current research was conducted, injuries were the primary cause of child death in 2021, accounting for 10,827 deaths and 2.15 million incident cases in children younger than 5 years of age [2-4].

Injury prevention frameworks such as the Haddon Matrix and the “4E” model have significantly shaped global policies and interventions over recent decades. Building upon these, the World Health Organization (WHO)’s World Report on Child Injury Prevention (2008) identified key interventions across many domains, including legislation, product safety, environmental modification, education, and emergency care, to reduce major types of child injuries [5]. High-income countries implement many of these strategies through structured programs such as The Injury Prevention Program (TIPP), which offers age-specific safety counseling and pediatrician training to enhance injury prevention practices [6]. However, such resource-intensive approaches require substantial institutional capacity, trained personnel, and long-term funding, posing implementation challenges for low- and middle-income countries (LMICs) such as China.

Chinese health authorities have incorporated injury prevention into the National Basic Public Health Service Program [7], a system whereby health-related information is conveyed to the population through health care providers. Despite its broad coverage, the program faces significant capacity constraints. Primary care providers are often understaffed and overburdened with routine services such as immunizations, chronic disease management, and regular medical examinations [8]. Most practitioners lack training in injury prevention, and the absence of operational guidelines limits implementation [9-11]. Consequently, neither globally recommended programs [5,6] nor technical guidelines [12] developed within China have been implemented in Chinese primary care settings.

Recognizing the potential of digital health as an alternative to deliver health-related messaging to parents and families, both the WHO and China’s national health strategies have emphasized the importance of integrating mobile health (mHealth) into public service delivery [13-15]. By leveraging digital technologies, mHealth can bridge gaps between research and practice by overcoming geographical and temporal barriers to deliver scalable, cost-effective interventions, including to resource-limited settings [16].

Further, the rapid advancement of artificial intelligence (AI) technologies has made it increasingly feasible to design interventions that integrate AI into mHealth apps. AI-enabled systems can ease workforce shortages by providing real-time support and interactions, automated risk assessments, and personalized recommendations. For example, a randomized controlled trial reported that an AI-powered digital platform for lifestyle change was effective in supporting weight loss among adults [17].

mHealth apps for child injury prevention have been piloted in high-income countries such as the United States [18-22], South Korea [23,24], and Australia [25], as well as in China [26-30]. Most of these interventions focus on delivering educational content via mobile platforms. However, several limitations have restricted their broader application in China [18-30]: (1) they primarily target preschool-aged children ages 3‐6 years, overlooking risk among infants and toddlers [18,19,21,24,27-30]; (2) they rely on standalone applications, creating limited user engagement and poor long-term adherence [18-29]; and (3) they typically require dedicated technical support, continuous user assistance, specialized intervention skills, and localized cultural and linguistic appreciation, infrastructure that is not embedded within the National Basic Public Health Service Program. Together, these limitations undermine scalability, sustainability, and integration into routine service delivery in China [18-30].

To address these gaps, we developed the Intelligent Child Unintentional-Injury Reduction & Education (iCURE) program that is integrated into China’s National Basic Public Health Service Program. iCURE is designed for health care providers to provide tailored advice on child injury prevention to caregivers via WeChat (Tencent), easing knowledge and temporal burden on providers and still conveying critical knowledge to caregivers. This protocol outlines a 12-month cluster randomized controlled trial to evaluate the effectiveness of iCURE in improving child injury–related knowledge, behaviors, and outcomes among caregivers of young children. If effective, iCURE offers a scalable and sustainable approach to incorporate child injury prevention into primary health care for young children and their parents across LMICs.

## Methods

### Study Design

A double-blind, 12-month, parallel-group cluster randomized controlled trial with 1:1 allocation ratio will be implemented in Changsha, Hunan Province, China. This study will strictly adhere to the SPIRIT (Standard Protocol Items: Recommendations for Interventional Trials) guidelines (Checklist 1)[31,32]. This protocol version is 1.0 and was enacted on August 1, 2024.

### Participant Recruitment

**Figure 1.**
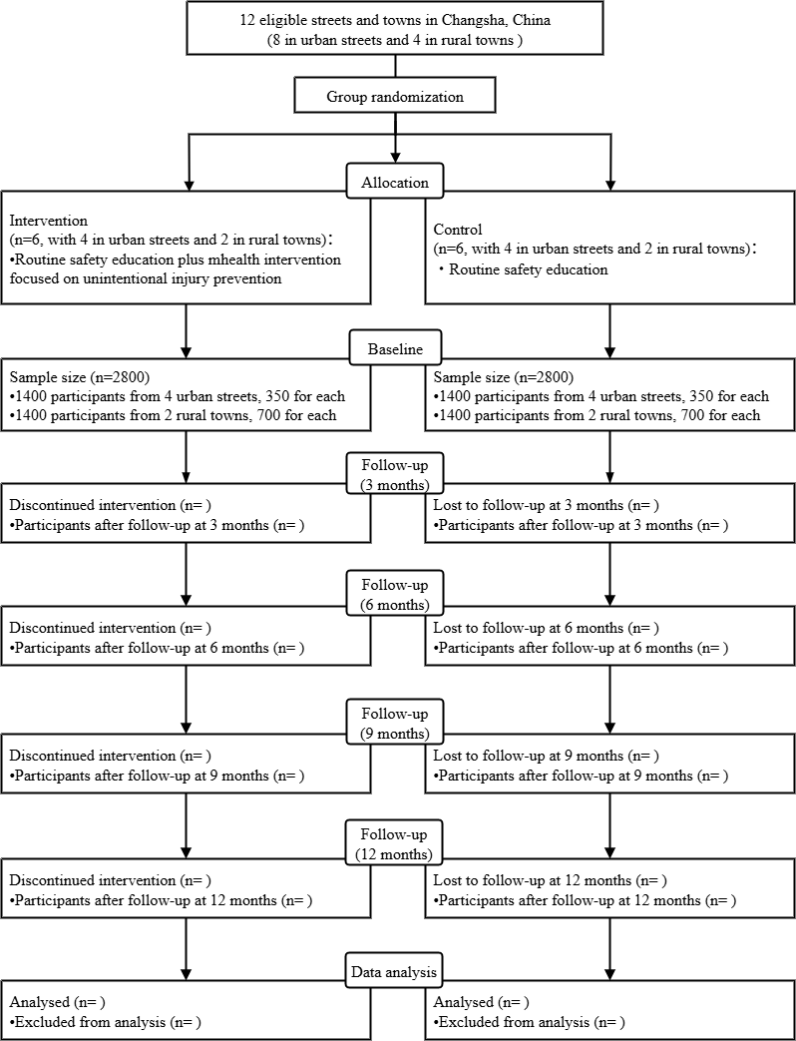
Study flow diagram.

A 2-stage recruitment strategy will be conducted. First, 12 streets and towns were selected from 5 administrative districts in Changsha, including 3 urban districts (Kaifu, Yuhua, and Yuelu) and 2 rural areas (Ningxiang and Liuyang). Each district consisted of at least 2 streets and towns that were eligible for study recruitment (Figure 1). Eligible streets and towns had a primary health care institution present that (1) provides basic public health services for a population with over 2000 children younger than 5 years of age and (2) reports they are not involved in any active child injury prevention programs.

One health care provider per institution will facilitate participant recruitment through (1) face-to-face communication, leaflet distribution, and poster display during routine services; (2) digital advertisements and instructional videos shared through established communication platforms, such as WeChat groups; and (3) collaboration with local schools serving children aged 3 to 5 years, where teachers will distribute flyers to caregivers.

Families will be eligible if children (1) are aged ≤5 years at the time of baseline survey; (2) live with a primary caregiver who provides daily support; (3) reside in the selected study site; (4) have a caregiver aged ≥18 years who is literate, owns a smartphone, and provides informed consent; and (5) are not enrolled in other structured injury prevention programs. Children with severe illnesses requiring specialized care or hospitalization will be excluded.

### Sample Size

Previous research reported a 12-month incidence of unintentional injury of 15.5% among children younger than 6 years of age in Changsha [33]. We hypothesize that the proposed mHealth intervention will reduce the injury incidence rate by 25% [34], with a design effect of 1.5 for the cluster sampling [35]. We also very conservatively assume 30% loss to follow-up [36]. Given these assumptions, a minimum sample size of 5178 children is needed to achieve 80% statistical power at a .05 significance level with a 1:1 allocation ratio between intervention and control groups (G*Power 3.1.9.7). Given that the actual values of certain parameters (eg, incidence rate and effect size) may be somewhat lower than assumed [28,34], we empirically expanded the sample size to recruit approximately 5600 children, with (2800 children from urban areas and 2800 from rural areas, including at least 350 children from each selected urban street and 700 from each rural town) within the constraints of available research resources. The 1:2 recruitment ratio of urban streets and rural towns is based on prior field experience, which suggests greater recruitment challenges in urban areas compared to rural ones.

### Intervention

The iCURE program is designed as an expert system, defined as a computer-based system that simulates the decision-making ability of a human expert, integrating the latest evidence-based knowledge on child injury prevention and applying rules of logic to provide context-specific guidance and tailored education to caregivers [37]. Its development is guided by expert system methodologies and the intervention mapping framework [38]. The design incorporates core principles from injury prevention and behavior change theories, along with empirical evidence and key stakeholders’ perspectives [39,40]. These components were integrated through a systematic development process involving literature review, needs assessment, and expert consultation [38]. The program applies algorithms such as keyword matching, text similarity analysis, decision tree models, and rule-based logic to deliver personalized recommendations and support [37,40-42].

A pilot study was conducted to assess the feasibility and operational performance of the iCURE program. The findings from the pilot were used to optimize its design and implementation, including improvements in system stability, registration flow, reward mechanisms, and data quality control.

### Key Components and Content of the iCURE Program

**Figure 2.**
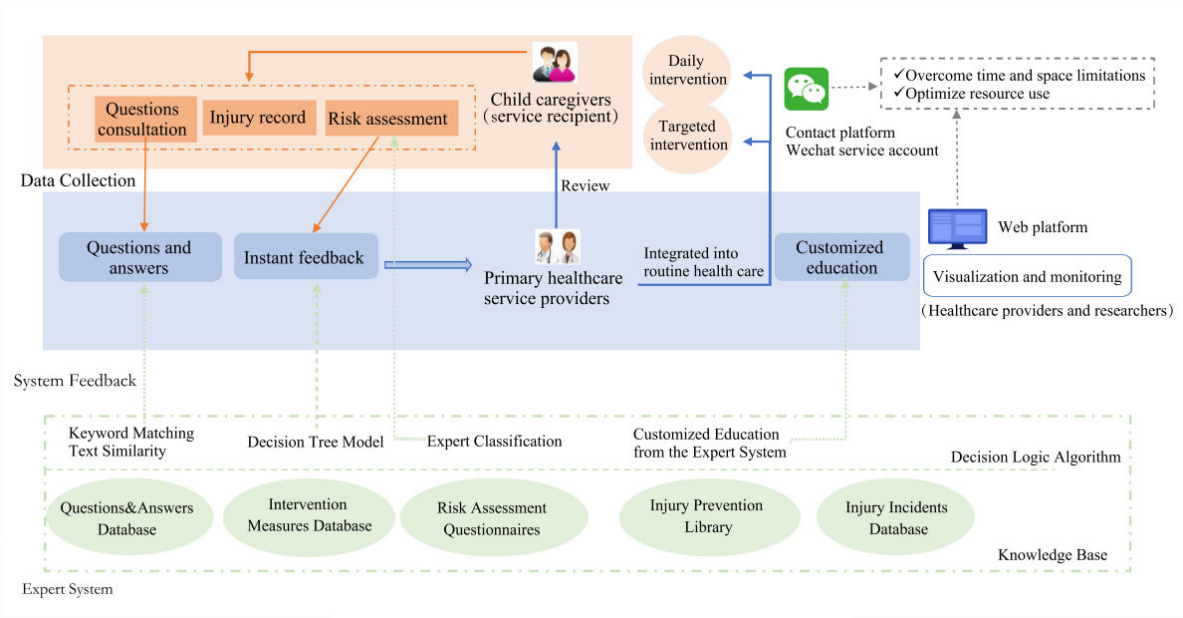
The functional modules and operational flowchart diagram of the mHealth intervention for child injury prevention.

The iCURE program consists of 2 components, a WeChat service account for caregivers and a web-based platform for health care providers (Figure 2 and MultimediaAppendices 1  2). This dual-platform approach facilitates information delivery and, crucially, supports caregiver-provider interaction.

The WeChat service account serves as the main channel for delivering intervention content to caregivers. The web-based platform is only accessible to health care providers, who support the system by managing and customizing educational content, monitoring caregiver engagement, and tracking self-reported injury events. All data are saved securely once collected and can be visualized on the platform. Providers and researchers can also export relevant data, including risk assessment outcomes, injury events, and intervention engagement, as needed (Multimedia Appendix 1).

The WeChat service account for caregivers comprises 5 key modules:

1. Injury risk assessment: a comprehensive child injury risk assessment questionnaire, developed based on validated tools [43-47], to assess child age, caregiver and child behaviors, and environmental risk.
2. Instant feedback: upon completing the risk assessment, participants receive immediate feedback and tailored recommendations generated by an integrated expert classification system and a decision tree model. This system stratifies children into low or moderate risk or high risk, which supports further personalized prevention strategies. For research purposes, participants who are randomly assigned to the control group will complete the assessment but will not receive feedback. Caregivers of children in the intervention group who are identified by the system as “high risk” will receive item-specific risk reports. This individualized risk profile will guide the dissemination of AI-tailored education materials for each child.
3. Injury reporting: caregivers are encouraged to report any injury events their children experienced during the study period. The module facilitates timely documentation and monitoring of injury events.
4. Interactive questions and answers (Q&A): caregivers may submit injury prevention questions through the platform’s real-time Q&A interface, which provides instant computer-generated and automated responses. The iCURE platform uses a hybrid natural language processing approach that combines keyword matching and text similarity analysis to retrieve and deliver relevant, automated responses from a curated injury prevention knowledge base created by the research group.
5. Tailored education: personalized educational content on child injury prevention is delivered in various formats (eg, text, images, long-form infographic post, flyers, and short videos). Delivery frequency is determined by a rule-based decision logic algorithm and tailored to the child’s risk level; caregivers of low-risk children receive content once per week, while those with higher-risk children receive it 2 or 3 times per week.

### Implementation Protocol

During routine consultations, primary health care providers will assist caregivers in registering for the iCURE WeChat service account. Upon registration, all caregivers will complete a questionnaire providing demographic information, their child’s injury history, and an injury risk assessment survey.

**Figure 3.**
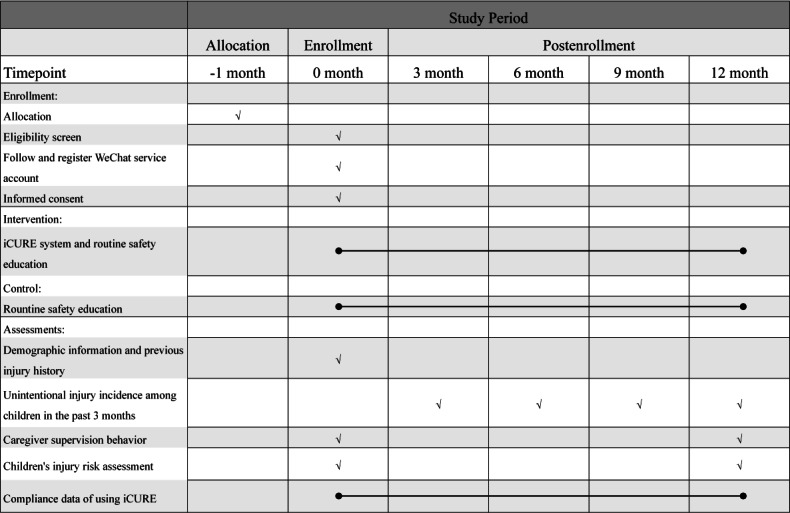
Timeline for the schedule of enrollment, interventions, and assessments. iCURE: Intelligent Child Unintentional-Injury Reduction & Education.

Caregivers randomly assigned to the intervention group will then receive the full version of the iCURE program, including real-time feedback, tailored educational content, and access to the Q&A module. The control group will access a simplified version involving completion of assessment surveys that are used only for data collection; they will receive no feedback, education, or supplemental materials. Both groups will continue to receive routine safety education provided by preschool teachers and primary health providers. Group allocation is detailed in Table 1. Details regarding the timeline for recruitment and intervention period are shown in Figure 3.

**Table 1.** Functions of iCURE (Intelligent Child Unintentional-Injury Reduction & Education) used by intervention group and control group.

Functions	Intervention group	Control group
Data collection	✓	✓
Injury risk assessment	✓	✓^a^
Instant feedback	✓	
Tailored education	✓	
Interactive questions and answers	✓	

a Control group will complete the injury risk assessment for data collection purposes, with no risk evaluation reports provided.

### Outcomes

The primary outcome is unintentional injury incidence, calculated as:


$$\frac{Number\;of\;children\;with\;injuries\;during\;the\;12-month\;study\;period}{Total\;number\;of\;children}\times 100\%$$

Injury events will be identified based on established criteria from previous studies [48], defined as any of the following: (1) receiving medical attention at a health care facility and being diagnosed with a specific injury; (2) receiving first aid from a nonmedical responder (eg, family member and teacher), such as wound dressing or bandaging; and (3) missing school for more than half a day due to an injury. We will also compare injury rates at 3-month intervals to assess the effect over time.

In addition, several secondary outcomes will be assessed:

(1) Change in child injury risk level: A 46-item questionnaire will assess children’s risk of unintentional injury, classifying children as low-to-moderate or high risk. The questionnaire was adapted from existing validated instruments and covers 8 common types of injuries: drowning (5 items), road traffic injuries (6 items), choking (6 items), falls (6 items), burns and scalds (6 items), poisoning (5 items), animal bites (5 items), and sharp and blunt object or foreign body injuries (7 items) [43-47].

(2) Change in caregiver supervision behavior score: Caregivers’ caregiver supervision behavior will be measured using a simplified version of a validated 5-item questionnaire, with responses rated on a 5-point Likert scale [49]. Items overlapping with the child injury risk assessment questionnaire were excluded to avoid redundancy.

### Randomization and Blinding

Randomization will be conducted at the street or town level to minimize potential contamination. Each street or town will be randomly assigned to either the intervention or control group, stratified by administrative division, using a random number sequence generated by R 4.4.1 software (R Core Team). The group allocation will remain concealed from both participants and the researcher conducting data analysis.

### Strategies to Enhance Engagement

To maximize caregiver engagement with iCURE, we will implement 4 strategies: (1) during registration, caregivers will be instructed to disable the “Do Not Disturb” mode in their WeChat service account; (2) caregivers in the intervention group will earn lottery entries for completing questionnaires and digital points for viewing educational materials, with both entries and points available to be redeemed in ranking competitions, as supported by prior research [28,50] and findings from a needs assessment; (3) safety-themed activities will be organized on designated special days, such as World Drowning Prevention Day, to reinforce injury prevention knowledge; and (4) iCURE materials will be shared through existing WeChat groups by teachers and health care providers in intervention areas to leverage community networks and expand outreach.

### Data Collection

Primary outcome data will be collected every 3 months to minimize recall bias. Secondary outcomes, which are less sensitive to recall bias, will be assessed annually. The baseline survey includes demographic information, child injury history over the past 12 months, caregiver supervision behaviors, and injury risk assessment, selected based on established associations with child injury and potential confounders [51]. All data will be self-reported, collected via the internet-based iCURE system, and securely stored on encrypted databases and hard drives. Caregivers will receive automated reminders followed by telephone follow-up if necessary to improve response rates.

The iCURE system will automatically record user engagement indicators, including (1) whether educational content was viewed, (2) frequency and duration of viewing, (3) messages to iCURE research team, and (4) online subscription to the iCURE WeChat service account.

### Data Analysis

Data will be analyzed following intention-to-treat principles and including all participants as originally assigned, regardless of adherence. Descriptive analysis (mean, SD, median, IQR, and proportion) will be used to describe baseline characteristics by groups. Differences in unintentional injury incidence across 5 timepoints (baseline, 3, 6, 9, and 12 mo) and other outcomes at 2 timepoints (baseline and 12 mo) will be examined using rank-sum tests for ordinal outcomes, chi-square tests for categorical outcomes, and independent 2-sample *t* tests for continuous outcomes. Within-group changes over time will also be evaluated.

Generalized linear mixed models with a Poisson link function will be used to compare the 12-month injury incidence between groups, adjusting for baseline covariates and accounting for clustering at the street or town level. Risk ratios and 95% CIs will be reported. Generalized estimating equations will also be used to assess repeated measures of injury incidence across follow-up timepoints, providing population-averaged estimates while accounting for within-subject and within-cluster correlations. Secondary outcomes, including changes in injury risk levels and caregiver supervision behaviors, will be analyzed using regression models appropriate to the type and distribution of the variable. User engagement indicators will be included as covariates of the effectiveness analysis model.

Two sets of sensitivity analyses will be performed to assess the robustness of findings: (1) per-protocol analyses and (2) models excluding participants with missing primary outcome data. Missing data will be documented and addressed using appropriate statistical methods as needed.

This study will adhere to the CONSORT (Consolidated Standards of Reporting Trials) 2025 [52] statement to perform data analysis and report results.

### Ethical Considerations

This study was reviewed and approved by the Ethics Committee of Xiangya School of Public Health, Central South University (XYGW-2024-71). All participants will provide electronic informed consent via the iCURE WeChat service account prior to participation, in accordance with national and institutional ethical guidelines. To ensure privacy and confidentiality, all data will be securely stored on encrypted databases and password-protected hard drives. Data will be de-identified before analysis, and no personally identifiable information will be retained in the analytic dataset. Participants will receive a modest digital monetary incentive ranging from 5 to 50 Chinese Yuan through a lottery-based mechanism via WeChat for their time and participation. No identifiable information will be included in the manuscript or any supplementary materials.

## Results

The study received funding in January 2023. Participant enrollment began in August 2024 and was completed in February 2025. A total of 6701 individuals completed baseline data, with 3110 allocated to the intervention group and 3591 to the control group (Table 2). The two groups somewhat differed in child age, place of residence, caregiver’s age, and education—the control group had higher proportions of children aged 3 to 5 years (69.7% vs 57.6%), residing in urban areas (61.3% vs 57.4%), caregivers aged 30 years and older (81.2% vs 74.9%) and receiving high or vocational school education and higher (85.7% vs 80.2%) than the intervention group (*P*<.05). The baseline 12-month injury incidence reported by caregivers was 8.86% (7.96-9.84) in the control group and 9.04% (8.06-10.11) in the intervention group.

By May 2025, 5,842 (87.2%) participants had completed the first follow-up survey.

**Table 2.** Number of recruited caregivers and children by demographic characteristics (%).

Variables	Overall, n (%)	Control, n (%)	Intervention, n (%)	*P* value
Total	6701 (100.0)	3591 (100.0)	3110 (100.0)	
Child gender				>.99
Girl	3147 (47.0)	1686 (47.0)	1461 (47.0)	
Boy	3554 (53.0)	1905 (53.0)	1649 (53.0)	
Child age (years)				<.001
1	1047 (15.6)	468 (13.0)	579 (18.6)	
1-2	1358 (20.3)	619 (17.2)	739 (23.8)	
3-5	4296 (64.1)	2504 (69.7)	1792 (57.6)	
Place of residence				.001
Urban	3988 (59.5)	2203 (61.3)	1785 (57.4)	
Rural	2713 (40.5)	1388 (38.7)	1325 (42.6)	
Caregiver’s gender				.09
Female	5400 (80.6)	2922 (81.4)	2478 (79.7)	
Male	1301 (19.4)	669 (18.6)	632 (20.3)	
Caregiver’s age (years)				<.001
＜30	1458 (21.8)	677 (18.9)	781 (25.1)	
30-39	4343 (64.8)	2408 (67.1)	1935 (62.2)	
≥40	900 (13.4)	506 (14.1)	394 (12.7)	
Number of children in the family				.51
1	3010 (44.9)	1627 (45.3)	1383 (44.5)	
≥2	3691 (55.1)	1964 (54.7)	1727 (55.5)	
Caregiver’s education				<.001
Junior high school or lower	1131 (16.9)	514 (14.3)	617 (19.8)	
High or vocational school	1493 (22.3)	816 (22.7)	677 (21.8)	
College or higher	4077 (60.8)	2261 (63.0)	1816 (58.4)	

## Discussion

### Anticipated Findings

This protocol outlines a 12-month cluster randomized controlled trial designed to evaluate the effectiveness of iCURE, an intelligent, WeChat-based mHealth intervention for child unintentional injury prevention. The intervention is structured to align with the current primary health care service model in China. If proven effective, iCURE can be generalized and implemented nationwide as a low-cost and easy-to-use unintentional injury prevention intervention.

Childhood injuries result in high morbidity and mortality rates worldwide, including in China. Although injury prevention has been integrated into China’s National Basic Public Health Service system, implementation has been limited due to workforce shortages, high workloads, inadequate training, and the absence of operational guidelines. This study addresses these gaps through the development and evaluation of iCURE. Similar mHealth initiatives have demonstrated effectiveness in child unintentional injury prevention [18-30]. However, unlike traditional app-based programs, iCURE operates via a widely used platform (WeChat) without requiring app installation and incorporates real-time, caregiver-reported injury monitoring. These features are particularly suited to resource-constrained settings and may support scalable implementation in LMICs.

The iCURE program adopts a structured and theory-informed design that is rooted in behavioral science and an injury prevention framework. By integrating an AI-assisted dissemination strategy that is guided by personalized risk assessment reports, it sends individualized and tailored education materials to each caregiver. Leveraging mHealth technologies, iCURE offers cost-effective, easily implementable, and real-time interactive solutions. In addition, the evaluation uses a rigorous cluster-randomized controlled trial design, with blinded outcome assessment and control for potential confounders. It includes clearly defined outcome measures and a standardized data collection process, enhancing the validity of findings.

Potential challenges to the study include inadequate caregiver enrollment, loss to follow-up, and reporting bias. To address these concerns, the study includes a multifaceted engagement strategy that includes incentives, safety-themed campaigns, and active follow-up via automated reminders and telephone outreach. The involvement of health care providers in participant recruitment and follow-up is crucial to maintaining engagement throughout the study. Data will be collected every 3 months to minimize recall bias.

To minimize potential recall bias from caregivers’ reports, we purposively adopted follow-up assessments every 3 months and strictly implemented standardized definitions of unintentional injury and prompts for minor events (eg, bandaging and disinfection). Nevertheless, such strategies cannot fully overcome recall biases as caregivers may interpret and treat injury events differently, and some might not report minor injuries that were not specified by the operational definition or were not perceived as injuries [53].

If proven effective, iCURE could enhance the capacity of China’s primary health care system to deliver standardized, accessible child injury prevention services. It also offers a practical model for integrating digital tools into national child health programs in LMICs, where injury prevention resources are typically scarce.

### Conclusions

This study responds to the urgent need for practical, evidence-based strategies to reduce childhood injury in China and other LMICs. Through a structured development process and rigorous evaluation, it aims to generate high-quality evidence on the effectiveness of an intelligent, system-integrated injury prevention program. If successful, iCURE could serve as a cost-effective, scalable solution for child injury prevention and surveillance, with potential for adaptation and dissemination through national public health platforms globally.

## Supplementary material

10.2196/76195 Multimedia Appendix 1 Home page of web platform.

10.2196/76195 Multimedia Appendix 2 Home page and modules of WeChat service account. Note: Eight images within the figure were derived from the WeChat service account “iCURE” (Intelligent Child Unintentional-Injury Reduction & Education) that was developed by the research team for unintentional injury prevention among children aged 0-6 and will be tested in this trial. (a) Home page of WeChat service account, (b) questionnaire filling interface, (c) injury risk assessment questionnaire, (d) feedback of risk assessment results, (e) feedback with tailored recommendations, (f) injury reporting module, (g) interactive Q&A (questions and answers) module, and (h) tailored education module.

10.2196/76195 Checklist 1 SPIRIT checklist

## Abbreviations

AI: artificial intelligence
CONSORT: Consolidated Standards of Reporting Trials
iCURE: Intelligent Child Unintentional-Injury Reduction & Education
LMIC: low- and middle-income country
mHealth: mobile health
Q&A: questions and answers
SPIRIT: Standard Protocol Items: Recommendations for Interventional Trials
TIPP: The Injury Prevention Program
WHO: World Health Organization
