# Supplementary figures and images for: Evaluating the Effectiveness of an Intelligent mHealth Intervention for Child Unintentional Injury Prevention: Protocol for a Cluster Randomized Controlled Trial
Source: JMIR Public Health Surveill. 2025 Jul 18;11:e76195. doi: 10.2196/76195 (PMC12296208; doi:10.2196/76195)

**Appendix 2**


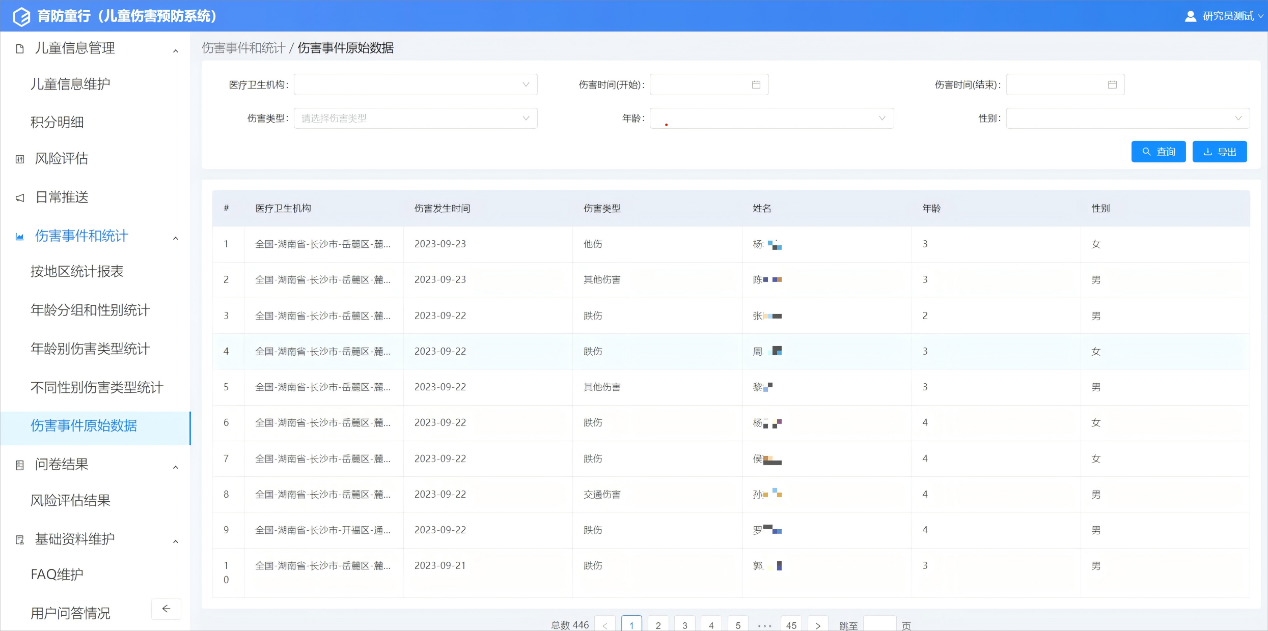


Figure 2 Home page of web platform.

Supplement: Multimedia Appendix 1 [file publichealth-v11-e76195-s001.docx]
