# Supplementary material for: Evaluating the Effectiveness of an Intelligent mHealth Intervention for Child Unintentional Injury Prevention: Protocol for a Cluster Randomized Controlled Trial
Source: JMIR Public Health Surveill. 2025 Jul 18;11:e76195. doi: 10.2196/76195 (PMC12296208; doi:10.2196/76195)
Supplement: Multimedia Appendix 2 [file publichealth-v11-e76195-s002.docx]

**Appendix 1**


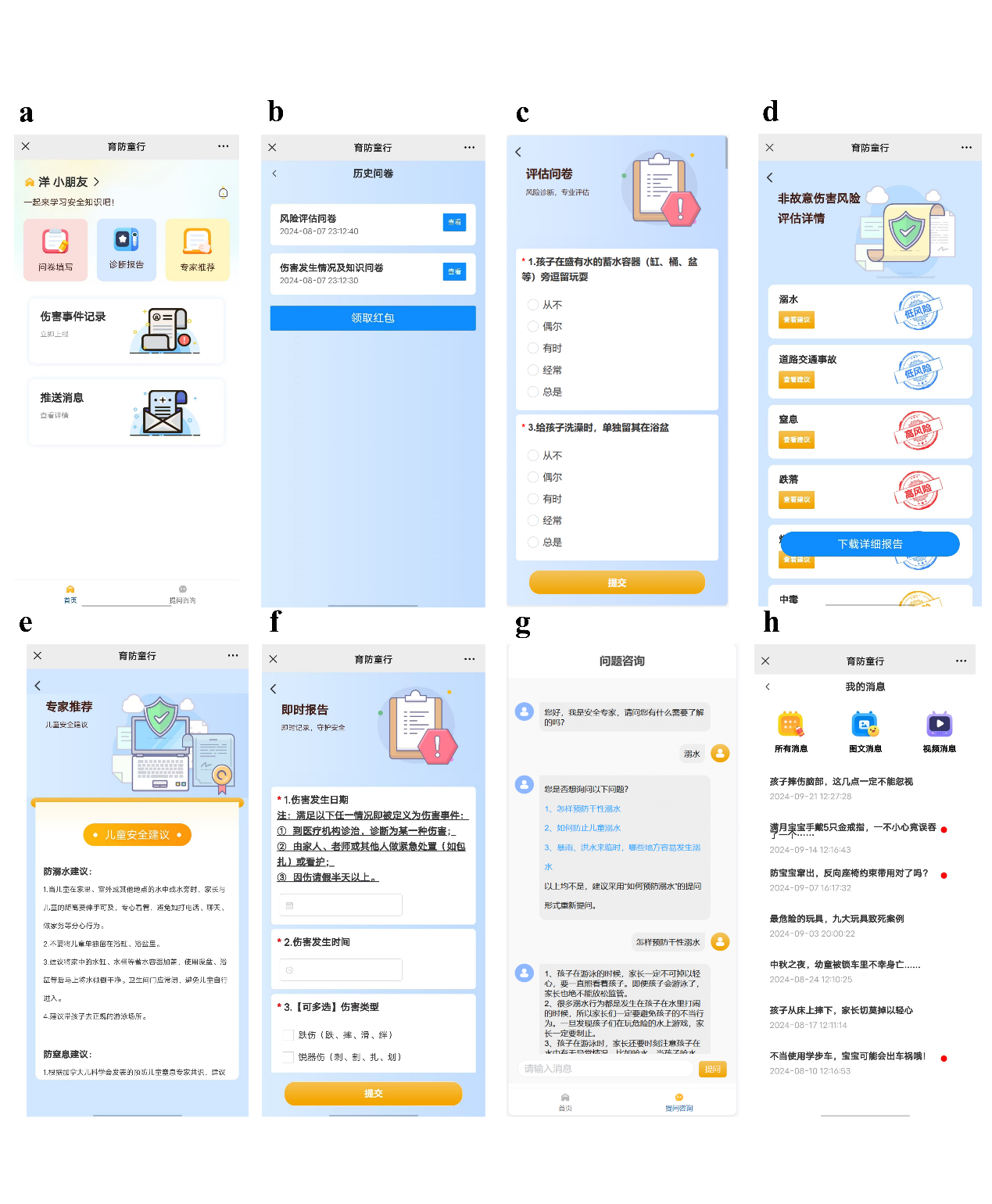


Figure 1(a-h) Home page and modules of WeChat service account. Note: Eight images within the figure were derived from the WeChat service account “iCURE” that was developed by the research team for unintentional injury prevention among children aged 0-6 and will be tested in this trial. (a) Home page of WeChat service account; (b) Questionnaire filling interface; (c) Injury risk assessment questionnaire; (d) Feedback of risk assessment results; (e) Feedback with tailored recommendations; (f) Injury reporting module; (g) Interactive Q&A module; (h) Tailored education module.
